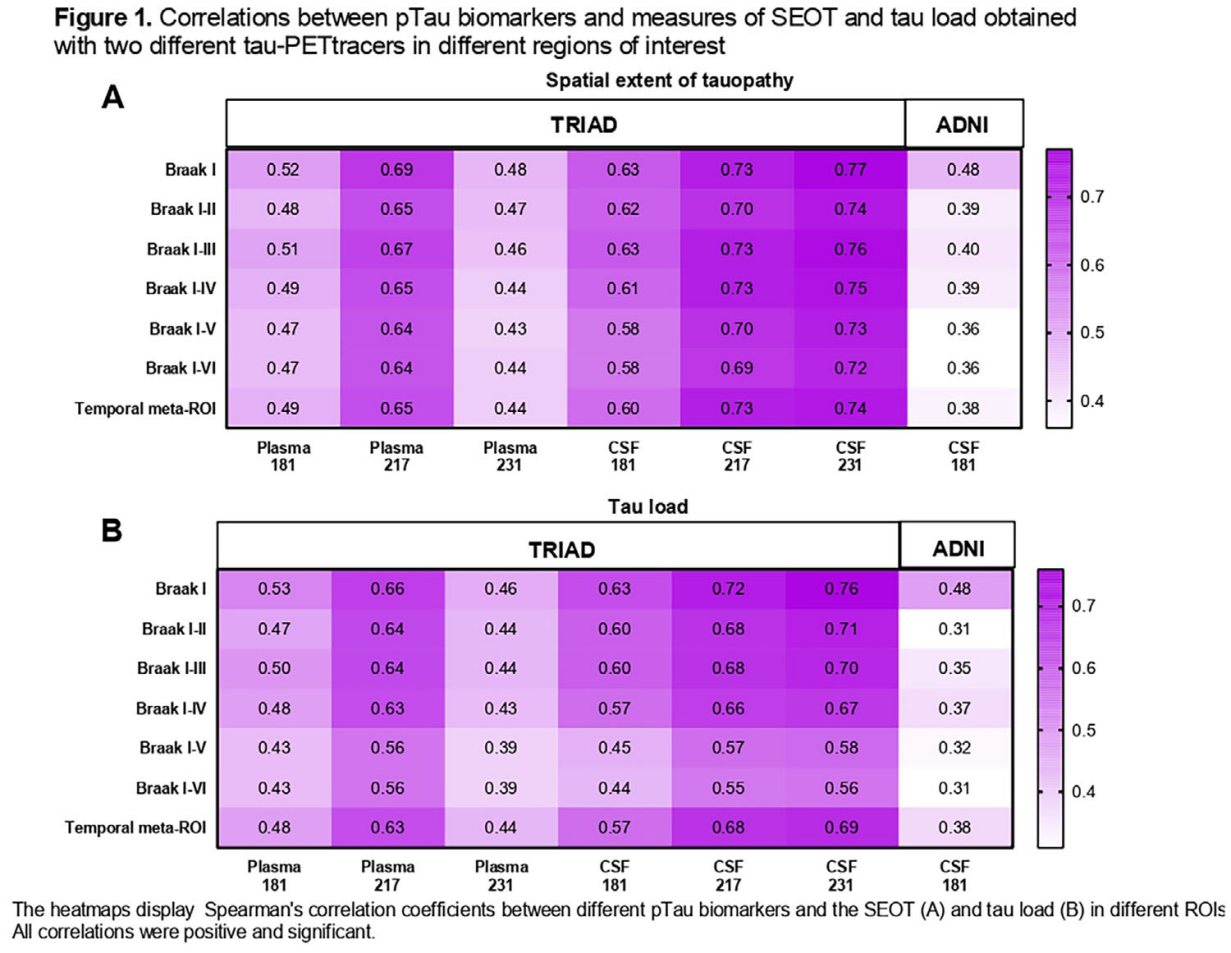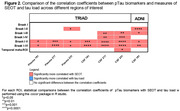# Phosphorylated tau is more closely associated with the spatial extent of tauopathy than with tau load

**DOI:** 10.1002/alz.093734

**Published:** 2025-01-09

**Authors:** Arthur C. Macedo, Nesrine Rahmouni, Joseph Therriault, Yi‐Ting Wang, Stijn Servaes, Cécile Tissot, Brandon J Hall, Jaime Fernandez Arias, Kely Monica Quispialaya Socualaya, Lydia Trudel, Firoza Z Lussier, Wagner Scheeren Brum, Eduardo R. Zimmer, Gallen Triana‐Baltzer, Hartmuth C. Kolb, Nicholas J. Ashton, Andrea L. Benedet, Thomas K Karikari, Kaj Blennow, Henrik Zetterberg, Tharick A. Pascoal, Pedro Rosa‐Neto

**Affiliations:** ^1^ Translational Neuroimaging Laboratory, The McGill University Research Centre for Studies in Aging, Montréal, QC Canada; ^2^ McGill University, Montreal, QC Canada; ^3^ Montreal Neurological Institute, Montreal, QC Canada; ^4^ University of Pittsburgh, Pittsburgh, PA USA; ^5^ Graduate Program in Biological Sciences: Biochemistry, Universidade Federal do Rio Grande do Sul (UFRGS), Porto Alegre Brazil; ^6^ Federal University of Rio Grande do Sul (UFRGS), Porto Alegre, RS Brazil; ^7^ Janssen Research & Development, LLC, La Jolla, CA USA; ^8^ Janssen Research and Development, San Diego, CA USA; ^9^ University of Gothenburg, Mölndal Sweden; ^10^ University of Gothenburg, Gothenburg Sweden; ^11^ University of Gothenburg, Mölndal, Gothenburg Sweden; ^12^ Departments of Psychiatry and Neurology, University of Pittsburgh School of Medicine, Pittsburgh, PA USA; ^13^ Translational Neuroimaging Laboratory, Montreal, QC Canada

## Abstract

**Background:**

Recent evidence indicated that cognitive impairment is more closely associated with the spatial extent of tauopathy (SEOT) than with tau load. It remains unclear whether this is also true for other markers of Alzheimer’s disease (AD) severity, such as fluid levels of phosphorylated tau (pTau). Here, we compared the link between fluid pTau and the SEOT and tau load in the brain, as assessed by PET.

**Method:**

We studied individuals across the aging and AD continuum or with non‐AD neurodegenerative diseases from the TRIAD and ADNI cohorts. TRIAD participants underwent [18F]MK6240 tau‐PET and CSF and plasma pTau 181, 217, and 231. ADNI participants were evaluated with [18F]AV1451 tau‐PET and CSF pTau181. We calculated standard uptake value ratios (SUVR) as a proxy of tau load, and the proportion of abnormal voxels as a measure of the SEOT. Abnormal voxels were those 2.5 SD higher than the mean of a group of young adults. Spearman’s correlations assessed the associations of pTau with SEOT and tau load in a temporal meta‐region of interest (ROI) and Braak‐like cumulative ROIs. Correlation coefficients were compared in R using the cocor package.

**Result:**

We included 325 participants from TRIAD (mean [SD] age, 68 [9.8] years) and 417 from ADNI (mean [SD] age, 71.8 [9.0] years). All pTau biomarkers showed significant correlations with SEOT and tau load in all ROIs (Fig. 1A‐B). All pTau epitopes were more closely associated with SEOT than with tau load in Braak I‐V and I‐VI (Fig. 2). In TRIAD, this was also observed in Braak I‐III and I‐IV for CSF pTau 217 and 231, and in Braak I‐II for plasma pTau 231. In ADNI, this was also seen for CSF pTau181 in Braak I‐II and I‐III. Tau load did not correlate better with pTau in any ROI.

**Conclusion:**

PTau biomarkers seem to inform better on SEOT than on tau load. There is a trend for better performance of SEOT as an imaging correlate of pTau when studying late Braak stages. Future studies should investigate the relationship between these measures and the clinicopathological progression of AD.